# Contact precautions prevent cross-contamination of extended-spectrum beta-lactamase-producing Enterobacterales in an intensive care unit: a prospective observational study

**DOI:** 10.1186/s13613-025-01589-y

**Published:** 2025-12-10

**Authors:** Nina Milerad, Christina Agvald Öhman, Inga Fröding, Christian G. Giske, Markus Castegren

**Affiliations:** 1 Department of Clinical Sciences and Technology (CLINTEC), Karolinska Institutet, Stockholm, Sweden; 2 Perioperative Medicine and Intensive Care (PMI), Karolinska University Hospital Huddinge, Stockholm, Sweden; 3 Department of Laboratory Medicine, Microbiology, Karolinska Institutet, Stockholm, Sweden; 4 Department of Microbiology, Public Health Agency of Sweden, Solna, Sweden; 5 Clinical Microbiology, Karolinska University Hospital, Stockholm, Sweden; 6 Centre for Clinical Research Sörmland, Uppsala University, Uppsala, Sweden

**Keywords:** Single room, Contact precautions, Extended-spectrum beta-lactamase-producing Enterobacterales (ESBL-E), Intensive care unit (ICU)

## Abstract

**Background:**

The spread of multidrug-resistant microorganisms (MDROs), including extended-spectrum beta-lactamase-producing Enterobacterales (ESBL-E), has increased worldwide and constitutes a significant public health challenge. International guidelines vary in their recommendations for isolation in single rooms and contact precautions regarding carriers of MDR microorganisms to mitigate cross-contamination in the ICU. The aim of this study was to investigate whether contact precautions without single-room isolation prevent cross-contamination of ESBL-E in an intensive care unit (ICU).

**Methods:**

All patients admitted to a general ICU during a period of 19 months were included. The study began before the COVID-19 pandemic and continued, albeit interrupted during the first wave, through the second and third wave. Rectal swabs, swabs from drainages and intravenous catheters were sampled for the detection of ESBL-E in all patients at the time of admission. Swabs were also taken from all patients co-treated with an ESBL-E-positive patient (i.e., the index patient) at the time of discharge. All cross-contaminated patient bacterial isolates were analyzed with whole-genome sequencing and compared to the isolate from the corresponding index patient.

**Results:**

Of 1042 patients admitted to the ICU, 82 patients were index patients, either known ESBL-carriers or tested positive at admission. 365 ESBL-E-negative patients (n=365) at ICU admission were co-treated in the same room as an index patient during their ICU-stay. Post-ICU discharge, three patients from the latter group tested positive for ESBL-E. No bacterial ESBLisolates from the latter patients corresponded to those of the index patients when their bacterial genomes were identified and compared.

**Conclusions:**

Contact precautions without single-room isolation of ESBL-E-positive patients did not result in any cross-contamination between ICU-patients in an endemic setting with a short length of stay.

## Background

The spread of multidrug-resistant organisms (MDROs), including extended-spectrum beta-lactamase (ESBL)-producing Enterobacterales (ESBL-E), has increased worldwide (Boucher et al. 2013). The prevalence of this pathogen is of major concern, as gram-negative bacteria account for a considerable proportion of hospital-acquired infections (HAIs), and the majority of the infections in the intensive care unit (ICU) [1]. Despite comprising a relatively small fraction of hospitalized patients, approximately 20% of all ICU patients have at least one HAI [2] during their stay. With an increasing number of colonized or infected patients, combined with development of antibiotics with new, unique mechanisms of action (Boucher et al. 2013), strategies to prevent the acquisition and cross-transmission of infections are crucial.

Swedish national guidelines and quality indicators for managing MDROs in ICU patients, effective until 1 January 2025, mandate single-room isolation for all MDRO carriers to prevent cross-transmission. CDC guidelines [3] recommend contact precautions for all patients colonized or infected with ESBL-producing organisms, as well as isolation in single rooms when available (CDC). In contrast, ESCMID guidelines (Tacconelli et al. 2014) recommend the same procedures, but only in epidemic settings (ESCMID). The ESCMID and CDC guidelines have mostly been studied in epidemic outbreaks, rather than in endemic ICU settings, suggesting that isolation in single rooms may be disputed in an endemic setting [4].

Risk factors for the acquisition of ESBL-E include a higher Simplified Acute Physiology Score version 3 (SAPS 3), intubation >48 h, as well as female sex and a history of previous antibiotic therapy [5–7]. Colonization, however, is a strong risk factor for infection [5, 6].

The principal aim of this study was to assess the degree of patient cross-transmission of ESBL-producing Enterobacterales in an ICU setting after implementing contact precautions for known ESBL-E carriers, replacing the previous routine of isolation of known ESBL-E carriers in single rooms..

The secondary aim was to investigate whether ESBL-E-carriage at admission was associated with any need for prolonged invasive intensive care measures or increased length of stay (LOS).

## Method

This prospective observational study was conducted in a 10-bed ICU at the Karolinska University Hospital, Huddinge. It is a tertiary/quaternary care hospital specializing in cell and organ transplantation, as well as patients undergoing extensive upper abdominal surgery, including liver, pancreatic, and esophageal surgery. Hence, there is a wide range of both medical and surgical ICU patients, including children. The ICU has three units, each with four beds, as well as three isolation rooms. Patients are normally cared for in four-bed rooms. The nurse-patient ratio is 0.8 with an assistant nurse-patient ratio of 0.6 [8].

All patients treated at the ICU were eligible for inclusion in the study. Exclusion criteria included patients who required isolation in single rooms for any other reason and those with missed admission cultures. Indications for single-room isolation included immunodeficiency, such as bone marrow transplant or other haematological diseases, or infection with Clostridioides difficile, Varicella-Zoster virus, Calici virus, Adenovirus, or carriage or infection with carbapenemase-producing Enterobacterales, MRSA (Methicillin-resistant Staphylococcus aureus), VRE (Vancomycin-resistant enterococci). All patients included in the study, including those colonized or infected with ESBL-E, were treated in four-bed rooms using contact precautions.

Rectal swabs, swabs from drainages or intravenous catheters were sampled in all patients at admission, and on all patients co-treated with an ESBL-E-positive patient at discharge. Hygiene measures for patients colonized or infected with ESBL-E were contact precautions, as pernational and international guidelines (Tacconelli et al. 2014), which included compliance with hand hygiene, wearing gown and gloves, and the use of disposable, single-use, non-critical care equipment. Environmental management was conducted in accordance with local protocols, involving daily cleaning of all surfaces surrounding the patient. Additionally, discharge cleaning was performed using disposable cleaning utilities, and all single-use equipment, including pillows, was discarded. Routine cleaning and disinfection protocols use detergent-based cleaning agents and disinfectants that incorporate surfactants [9].

The study was planned to proceed for one year, however, it was extended due to the SARS-CoV-2 pandemic. The study began before the pandemic but suffered from an extreme need to relocate resources during the first wave of the pandemic in the Stockholm area (20 March 2020–31 August 2020). Consequently, the study was paused during the first wave and resumed thereafter.

Age, sex, comorbidities, height, and weight were recorded at the time of admission. Additional patient data from the ICU stay and treatment were extracted from the electronic medical records. One research nurse collected all dataat admission, discharge and follow up.

### Ethical statement

Study information was readily available to all patients and their families in the ICU. The study was approved in advance by the Swedish Ethical Review Authority (dnr:2019-04576). The ethical permit stated that informed consent was not needed for inclusion to the study.

### Microbiological analysis

ESBL-E colonization was defined as either a known ESBL-E carrier, irrespective of positive or negative screening at admission, or an unknown carrier with positive screening at admission.

A new acquisition of ESBL-E was defined as a negative screening result at admission, followed by a positive screening at discharge.

Patient-to-patient transmission was examined using the following steps: a known carrier, or a newly identified patient positive for ESBL-E at admission, was designated as the index patient. All patients co-treated in the same room were identified, and screening samples were collected at discharge, in addition to the admission samples. If any of the discharge samples from the identified patients were positive, whole genome sequencing (WGS) of the bacterial isolate was performed, and bioinformatic comparison of the strains was conducted, thereby either verifying or refuting the possibility of cross-contamination between patients treated in the same room as the index patient. Cross-contamination was confirmed when ESBL strains and WGS from a new acquisition suggested a close genetic relationship with the strain from the index patient. Cross-contamination was ruled out when WGS analysis indicated low genetic relatedness between acquired strains and the strain from the index patient.

### Bacterial cultures

Bacterial cultures were performed at the Karolinska University Laboratory using routine diagnostic procedures. Rectal swabs (Sigma-Transwab, MWE, Corsham UK) were plated on in-house chromogenic ESBL-screening agar (Chromatic ESBL agar base, Liofilchem, Roseto degli Abruzzi, Italy, with cefpodoxime 6 mg/L) and carbapenemase screening agar (Chromatic ESBL agar base, Liofilchem, Italy, with meropenem 0.25 mg/L and cloxacillin 200 mg/L) and incubated for a minimum of 18 h. Colonies were identified at the species level using MALDI-TOF/MS (Bruker Daltonics, Billerica, MA). Antimicrobial susceptibility testing was performed according to the European Committee on Antimicrobial Susceptibility Testing (EUCAST) disk diffusion method [10] or VITEK2 (bioMérieux, Marcy-l’Étoile, France). Identification of ESBL-production was performed using VITEK2, AST double disk synergy test [1], and/or ESBL/pAmpC diagnostic kit from ROSCO Diagnostica A/S (Taastrup, Denmark). Carbapenemase-production was tested using KPC/MBL and OXA-48 Confirm kit 98015 from ROSCO Diagnostica A/S. The presence of carbapenemase- and pAmpC-genes was confirmed with Eazyplex SuperBug kits (Amplex Diagnostics GmbH, Gars-Bahnhof, Germany). Enterobacterales isolates with evidence of ESBL- and/or pAmpC were defined as ESBL-producing Enterobacterales in accordance with the case definition for the Swedish Public Health Agency [11].

### Whole-genome sequencing of bacterial isolates

Extraction of genomic DNA was performed with the EZ1 Advanced XL system (Qiagen, Hilden, Germany). The quantity of the extracted DNA was measured using a Qubit double-stranded DNA (dsDNA) assay kit (Life Technologies Europe). Extracted DNA was sequenced on Illumina HiSeq 2500 at the Science for Life laboratory (SciLifeLab, Solna, Sweden), generating 2 × 100 paired-end sequences. Assembly of raw reads and multilocus sequence typing (MLST) was performed through the in-house bioinformatic pipeline BactTyper at the Public Health Agency of Sweden. The pipeline filters and assembles genomic reads using CLC assembly cell version 5.2.1 (QIAGEN, Hilden, Germany). Resulting assemblies are compared to reference genomes in our in-house database using BLASTn to determine the species or species complex [12]. Curated agent databases were used to annotate the assembly and identify AMR genes and MLST, using a BLAST and mapping approach to produce relevant coverage and identity data. A SNP analysis of isolates of the same ST was performed by aligning the genome to an in-house reference for the sequence type (ST), performing a variant call, and reconstructing a phylogenetic tree using FastTree2 [13]. We also produce a minimum spanning tree (MST) using MSTgold, with SNP distances as edge weights [14].

### Statistical analysis

Age, sex, SAPS 3 score, invasive treatments administered, length of stay at the ICU, and mortality in the ICU data were retrieved for each patient. Data are presented as proportions or as medians with interquartile ranges. Intergroup differences were analyzed using t-tests or the Mann Whitney *U* test, depending on the data distribution. Fisher´s exact test was used for differences between proportions. Statistica™, version 13.5 (TIBCO Software Inc., Palo Alto, CA, USA) was used for the statistical analyses. A p-value of <0.05 was considered statistically significant.

## Results

The study started 4 November 2019 and ended 30 June 2021. It was paused during the first wave of the SARS-CoV-2 pandemic, from 17 March 2020 to 31 August 2020, and again from1 January 2021 to 22 January 2021, because of the renewal of the Ethics Review permit.

**Fig. 1:**
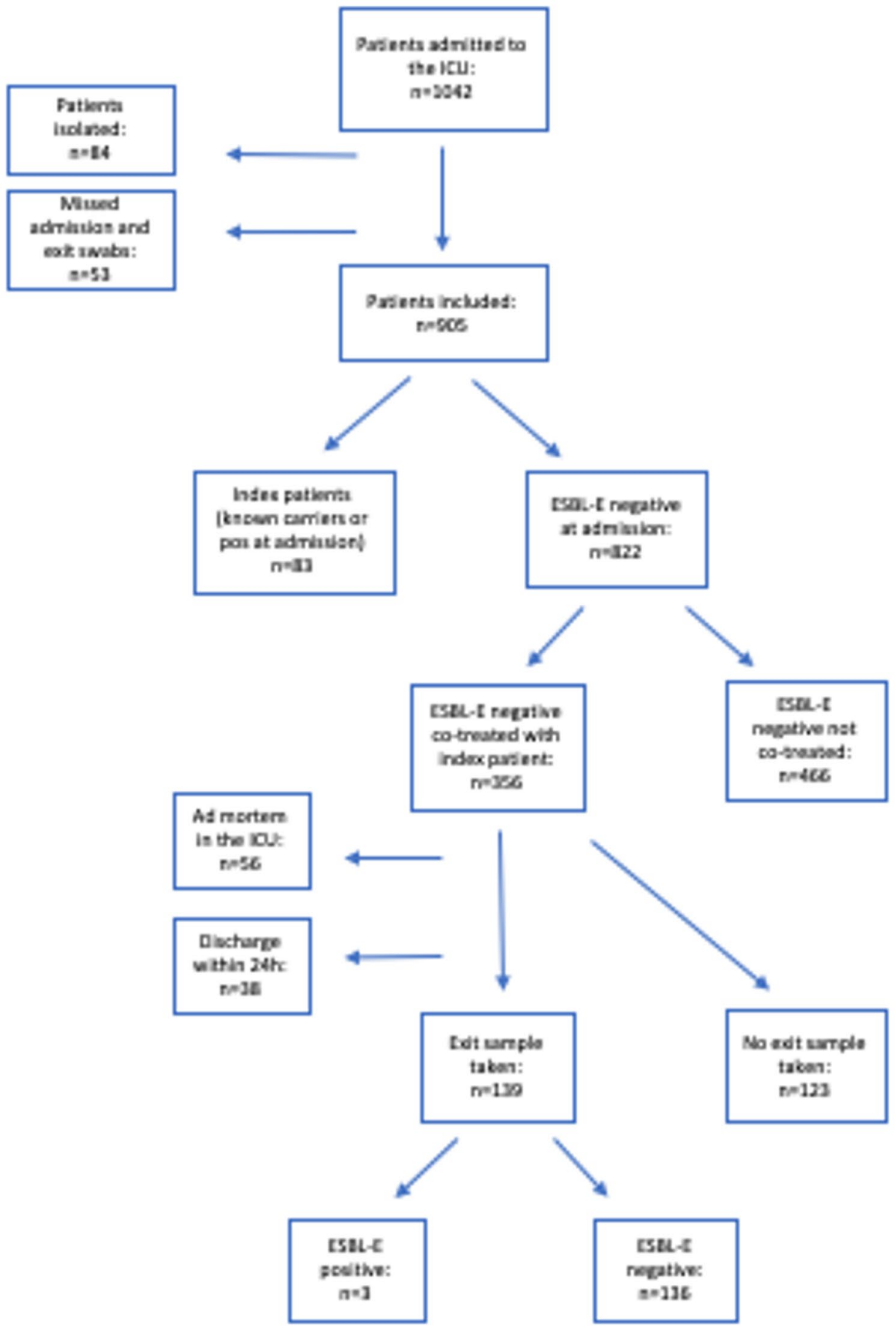
Flowchart of patients included in the study. *ESBL-E* extended-spectrum beta-lactamase-producing Enterobacterales, *ICU* intensive care unit

A total of 1042 patients were admitted to the ICU during the study period (of which 129 were children <20 years of age). Figure 1 depicts the consort diagram of the study. Of all patients included in the study, 84 (8%) were excluded because of isolation requirements, and 53 (5.5%) were excluded because of missing admission swabs. A total of 905 patients met the inclusion criteria. Of those patients, 83 (9%) were known ESBL-E- carriers or were ESBL-E positive at the admission screening. 822 patients were ESBL-E-negative at admission, of whom 356 were co-treated with an index patient during their ICU stay. Of the co-treated patients, 139 had exit samples taken according to protocol. Of the missing exit samples, 56 were unavailable because of patients deceased in the ICU, 38 resulted from discharges within 24 h, and the remainder were either improperly collected or lost. Three patients were ESBL-positive at discharge and were considered new acquisitions.

ESBL-E-status at admission did not show any effect in demographic data, invasive treatment received, LOS or ICU mortality, see Table 1.

**Table 1:** Demographics of patients, treatment received, and mortality

	All ICU-admitted patients	Patients included in the study (n=905)			
		Index patients (positive at admission)	Co-treated (negative at admission and ESBL-E exit sample taken)	p*	New acquisitions
n (%)	1042 (100)	83 (9)	139 (15)	N/A	3
Female sex n (%)	400 (38.4)	27 (32.5)	50 (36)	0.6	2 (66.7)
Age (y)	61 (41–71)	56 (37–68)	59 (44–72)	0.11	56 (39–66)
SAPS 3	52 (39–63)	50 (37–58)	52 (42–62)	0.83	53 (51–53)
Antibiotic treatment n (%)	450 (43.2)	35 (42.1)	75 (54)	0.09	2 (66.7)
Invasive ventilation n (%)	568 (54.5)	43 (51.8)	83 (59.7)	0.25	2 (66.7)
Time on ventilator (h)	87.3 (21–194)	94.6 (50–169)	96.2 (21.3–169.9)	0.6	125 (8–242)
CRRT n (%)	22 (2)	1 (1.2)	3 (2.2)	0.59	0
Time on CRRT (h)	29.6 (12.8–74)	189.5	74.1 (12.2–119.5)	0.28	N/A
LOS ICU (d)	1.75 (0.6–5)	2.79 (0.7–6.2)	2.13 (0.85–5.5)	0.65	3.5 (3.2–7.6)
ICU mortality n (%)	133 (12.8)	7 (8.4)	57 (16)	0.077	0

Values are presented as proportions or medians with the interquartile range in brackets. The p-values are results from t-tests analysis or Mann-Whitney *U*-test where appropriate and Fisher’s exact test comparing proportions

SAPS 3-simplified acute physiology score, version 3; RRT-renal replacement therapy; LOS ICU-length of stay in the intensive care unit

*p denotes results comparing patients ESBL-negative at admission with ESBL-positive at admission

### Microbiological analyses and whole-genome sequencing

The most common ESBL-producing bacteria was *E. coli*, followed by *Klebsiella pneumoniae* (Table 2).

**Table 2:** Species, sequence types (ST) and ESBL/pAmpC groups for index cases^a^ (n = 55 isolates from 51 patients accounting for 60 episodes) and patients with acquired ESBL^B^ (n=3)

Species	Index cases^A^							Acquired ESBL^B^		
	n=	CTX-M-1 group	CTX-M-27 group	CTX-M-9 group	TEM-ESBL group	CMY/DHA (pAmpC)	CTX-M-1 group + CMY	n=	CTX-M-1 group	CTX-M-9 group
*Escherichia coli*	49	28	8	5	1	6	1	2	1	1
ST131	10	7	3					0		
ST69	6	2	1	2		1		1	1	
ST38	4	1	1	2				0		
ST10	3	2				1		0		
ST127	3	3						0		
ST405	3	2					1	0		
Other STs	20	11	3	1	1	4		1		1
*Klebsiella pneumoniae*	5	2		2		1		0		
*Proteus mirabilis*	1					1		0		
*Salmonella enterica* ser. Typhimurium	0							1		1
Total	55	30	8	7	1	8	1	3	1	2

^a^Known carriers or ESBL culture positive at admission

^b^Acquired ESBL i.e. co-treated with index patient with positive sample at discharge

Note. Bacterial isolates were not available from known carriers in 23 episodes (21 patients) due to negative or missed admission cultures

The incidence of ESBL-E acquisition among the ESBL-E-negative patients co-treated with an ESBL-E-positive patient was 2%. Of the three acquisitions, two were caused by *E. coli* and one was caused by *Salmonella enterica serovar Typhimurium*. The genomic sequencing of the bacterial isolates of the new acquisitions did not provide any evidence for cross-transmission during the study.

## Discussion

Contact precautions i.e. compliance to hand hygiene, wearing gowns and gloves, and the use of disposable, single-use, non-critical care equipment, without the use of single rooms for isolation of ESBL-E-positive ICU patients, did not lead to any case of bacterial, genomic-identical cross-contamination to patients co-treated in the same room in our study. Only three cases of new ESBL-E acquisitions were identified, with no proven cross-transmission.

Our results align with previous studies, demonstrating minimal cross-transmission in both ICU and non-ICU settings. Repessé et al. found an incidence of ESBL-E acquisition of 4.1% with only two cases of cross-transmission among 470 patients in a 12-bed French ICU with no single rooms [4]. The prevalence of ESBL-E carriage was 13.2%. In a large tertiary care hospital in Switzerland, Tschudin-Sutter et al. identified only two cases of cross-transmission among 133 ICU and non-ICU patients, resulting in colonization without infection [15]. In a French medical ICU, Alves et al. confirmed only one cross-transmission among 19 acquisitions in the 309 patients included in the study [16]. ESBL-E carriage at admission was reported in 8% of the patients. Our study identified ESBL-E carriage in 9% of the admitted patients, which is comparable to the findings of Alves and Repessé.

We acknowledge several limitations of our study. First, this was an observational study conducted in a single center ICU. Second, a significant number of discharge swabs were missing or incorrectly collected, mostly at the study´s conclusion. This period overlapped with the second and third waves of the SARS-CoV-2 pandemic in the Stockholm region. Although the study was paused during the first wave of the SARS-CoV-2 pandemic, which hit the Stockholm region the hardest, during the second and third waves the ICU expanded from a 10-bed ICU to a maximum of 30 ICU beds, resulting in a decrease in the nurse-patient ratio from 0.8 to 0.5. Due to a continuous high pressure on ordinary ICU staff, combined with occasional inexperienced personnel, discharge routines were occasionally not maintained, resulting in missed or incorrectly taken discharge swabs. The high nurse-patient ratio of 0.8 during normal conditions decreased substantially during the second and third waves, thereby increasing the risk of acquisition.

An additional limitation in this study is the short median length of stay. The brevity of hospital stays may be partially attributed to the SARS-CoV-2 pandemic, which coincided with a decline in other patient groups with diagnoses such as pneumonia and influenza, as well as elective surgery being canceled [17]. Alves et al. showed that patients who acquired ESBL had a significantly longer ICU stay; thus, a shorter median LOS at our ICU might be associated with the lower acquisition rates. In our study, the median LOSamong index patients were 2.8 days, compared to 7 days reported by Alves et al. and 13 days reported by Barbier et al. [16, 18]. The median LOS in Swedish ICUs during 2024–2025 was 2.51 days[19]. This reduces the generalizability of the results when applying them to other ICUs with longer LOSs.

Based on the discharge samples collected, we observed fewer new acquisitions compared to the studies mentioned above. Aside from the obvious risk of missed acquisitions due to missed discharge swabs, another explanation might be that we accounted for index patients as both those with positive admission samples and known carriers, even though they had negative admission samples. This procedure may result in fewer acquisitions per index patient.

The acquisition of ESBL-E in the ICU is multifactorial; the contact precautions assessed in this study are only one of several factors. Other factors include adherence to hand hygiene, environmental hygiene measures, safe handling of excreta, and control of antibiotic therapy (antibiotic stewardship). Adherence to hand hygiene was not collected during the study period; however, it was about 65–70% when measured from 2022 to 2025. Environmental hygiene protocols, as specified in the methodology, were strictly adhered to.

Pevious exposure to antibiotics might also affect acquisition. In a study by Tacconelli et al. [20], antibiotic therapy during hospital stay was associated with an increased risk of acquiring ESBL-E up to seven days after treatment. The most significant effect was observed with monotherapy using cephalosporins, while combination therapy had a slightly reduced risk compared to monotherapy. Antibiotic use in all patients admitted to the ICU was 43.2%, but data on antibiotic treatment before admission to the ICU was missing. Because most infections acquired in the ICU are endogenous from the patient´s microbiome, rather than exogenous infection, another explanation for fewer new acquisitions in this study compared to other studies might be the low prevalence of ESBL-E in Sweden (4.7% ESBL-carriage was reported in a 2012–2013 nationwide Swedish study [21]), compared to other European countries, leading to a lower colonization pressure. Colonization pressure refers to the prevalence of bacteria that are resistant to antibiotics in the environment. When prevalence is high, the risk of acquisition increases due to an increased risk for environmental contamination and higher rates of transient hand carriage among individuals (e.g., health care workers). The importance of colonization pressure has been measured for VRE and MRSA [22], but no such estimation has been made for Gram-negative bacteria. 

This study has several merits. We defined all patients with previously known ESBL-E-carriage as carriers, even if they had negative screening samples at admission. This was done to minimize the risk of missing any cross-contamination. Fecal carriage often persists for one year after acquisition or infection, and negative fecal samples do not necessarily indicate end of carriage [23].

Is cross-transmission between co-treated patients the primary mode of ESBL-E acquisition? Our study implies the opposite. Other studies suggests that patient-to-patient transmission may not be the only significant cause of acquiring ESBL-producing *E. coli* in the ICU [24]. Contamination from health care workers hands might be one factor contributing to transmission [3], as may contaminated medical products, dry surfaces, or moist environments [25]. Although contact isolation is often recommended for all MDROs, there is a diversity in spreading patterns between various bacterial species. Multidrug-resistant Gram-positive organisms, such as methicillin-resistant *Staphylococcus aureus* (MRSA) and vancomycin-resistant enterococci (VRE), can, for example, survive days or weeks on surfaces, in contrast to Enterobacterales [26]. This observation suggests that recommendations may vary depending on the specific MDRO.

Beyond the obvious economic importance of using ICU resources and staff as effectively as possible, isolation of patients in single rooms is also associated with an increased risk of adverse events and reduced contact with health care workers [27]. The benefits of isolating patients should therefore outweigh the risks.

## Conclusions

This study suggests that contact precautions without single-room isolation may prevent cross-contamination of ESBL-producing Enterobacterales among ICU patients in an endemic setting with a short LOS.

Tailoring recommendations to the specific MDRO strain and endemic or epidemic ICU setting may offer a more constructive and cost-effective approach to reducing new acquisitions of ESBL-E during intensive care.

## Data Availability

The datasets used and/or analyzed during the current study are not publicly available to protect patient privacy.

## Abbreviations

**ESBL**: Extended-spectrum beta-lactamase
**ESBL-E**: Extended-spectrum beta-lactamase-producing Enterobacterales
**MDRO**: Multidrug-resistant microorganisms
**ICU**: Intensive care unit
**SAP****S ****3**: Simplified acute physiology score version 3
**MRSA**: Methicillin-resistant *Staphylococcus aureus*
**VRE**: Vancomycin-resistant enterococci
